# Common marmosets show social plasticity and group-level similarity in personality

**DOI:** 10.1038/srep08878

**Published:** 2015-03-06

**Authors:** Sonja E. Koski, Judith M. Burkart

**Affiliations:** 1 Anthropological Institute and Museum, University of Zürich, Winterthurerstrasse 190, CH-8057 Zürich, Switzerland

## Abstract

The social environment influences animal personality on evolutionary and immediate time scales. However, studies of animal personality rarely assess the effects of the social environment, particularly in species that live in stable groups with individualized relationships. We assessed personality experimentally in 17 individuals of the common marmoset, living in four groups. We found their personality to be considerably modified by the social environment. Marmosets exhibited relatively high plasticity in their behaviour, and showed ‘group-personality’, i.e. group-level similarity in the personality traits. In exploratory behaviour this was maintained only in the social environment but not when individuals were tested alone, suggesting that exploration tendency is subjected to social facilitation. Boldness, in contrast, showed higher consistency across the social and solitary conditions, and the group-level similarity in trait scores was sustained also outside of the immediate social environment. The ‘group-personality’ was not due to genetic relatedness, supporting that it was produced by social effects. We hypothesize that ‘group-personality’ may be adaptive for highly cooperative animals through facilitating cooperation among individuals with similar behavioural tendency.

Animal personality is defined as substantial variation among individuals’ behaviour patterns, which is consistent through time and contexts^1^,^2^. Although consistency is the main criterion of personality, it can vary between species, populations, and individuals, because plasticity is subjected to natural selection^3^,^4^. Fundamentally, behaviour is regulated by physiological mechanisms, which themselves are regulated by genetic and epigenetic factors^5^,^6^,^7^. However, the more plastic behaviour is, the more it can be influenced also by external effects, involving developmental^8^, ecological^9^,^10^, or social^11^ factors.

Social environment can influence personality in various ways. Sociality may favour behavioural consistency and differentiation of behavioural types through social niche specialization^12^,^13^ (but see Ref. 14), resulting in differences in personality trait levels within a social group. Social environment may also push individuals’ personality towards similarity by e.g. social facilitation or conformity^11^,^15^. Furthermore, individuals may respond to the social environment by short-term increase or decrease of trait expression. For example, exploration behaviour^16^ and activity^17^ can increase when individuals are tested socially compared to being tested alone, and these effects can be sex- or relationship-specific^11^.

The ways in which the social environment influences personality in highly social species is, however, poorly understood. Many species live in groups with stable and long-lasting, individualized relationships. In such an environment, personality is manifested by default in the social environment and thus, may be subjected to constant social effects. Social effects on highly plastic behaviour may even lead to group-level similarity in personality, akin to cultural behaviour^18^,^19^. Group differences in social personality traits, not explicable by ecological or demographic factors, were found in captive chimpanzees^20^. Recently, groups of semi-wild chimpanzees living in identical ecological conditions were shown to vary in their social behavioural “styles”^21^.

We assessed the effects of the social environment on personality in common marmosets (*Callithrix jacchus*). Marmosets, like many other primates, are highly socially cohesive, which makes social modification of their personality possible. We addressed social effects on the universal personality traits of boldness and exploratory tendency^1^. Although threat and novelty responses are assumed to be mainly regulated by internal mechanisms^5^, social modification in them has been described^15^,^16^. In addition, we measured individual variation in persistence, because it may correlate with exploration tendency in species that rely on social learning^22^.

We expected marmosets to show social effects on the personality traits for three reasons. First, primate behaviour is generally plastic and vulnerable to social influence. Differences in the ecology and social conditions cause within-species differences in the social systems^23^,^24^. Experimental transfers of individuals between two closely related species with different social dynamics show that the social environment can influence behaviour enough to result in species-atypical levels of aggression and affiliation^25^. Moreover, there are group differences in behavioural styles that are not directly connected to the surrounding ecology, but result from learned behavioural patterns and are referred to as culture^18^,^26^. In some cases, conformity to others’ behaviour has been experimentally shown^27^. Second, social learning modifies marmoset foraging behaviour^28^,^29^,^30^, which may be associated with exploratory tendency. Third, marmosets are cooperative breeders, that is, groups consist of a breeding pair and adult offspring that forgo breeding for remaining in the natal group as adult helpers. Cooperative breeding requires coordinated cooperation and keen attentiveness to others’ behaviour^28^. Therefore, cooperative breeding may make individuals particularly attuned to others' behaviour and likely to adjust own behaviour accordingly.

Intriguingly, our study revealed group differences in marmoset personality, in that members from the same social group had more similar personality trait scores than individuals from different groups. We explored three mechanisms that may be responsible for this ‘group-personality’. (i) Genetic factors, when strongly canalized traits are similar among related individuals and groups have a high degree of relatedness. In this case, genetically close individuals should be more similar to each other, even if in different groups, than unrelated individuals, even within the same group. (ii) Long-term social influences in the early development or after immigration through some learning mechanism^19^. In this case, group members should show consistent behavioural similarity with each other independent of the presence or absence of group members. (iii) Short-term social effects, such as social facilitation, on highly plastic traits. In this case, group members should show behavioural similarity only in the presence of each other, but not when tested solitarily.

As this is the first targeted study on marmoset personality, we quantified repeatability^31^ regarding both temporal and cross-situational consistency in the target traits. This is the crucial basic step to assess whether the traits meet the formal criteria of personality. To do so, we conducted experiments in a social setting, because marmosets are obligate group-living animals and thus express their normative behavioural range in a social environment^22^. To address the social effects such as facilitation or competitive exclusion on personality, we conducted a subset of the experiments in a solitary condition. In particular, we investigated whether repeatability of behaviour was dependent on the social environment. We also assessed whether sex or social role (i.e. breeding status) predict trait scores. Sex differences in exploratory behaviour were expected because females have been reported to be faster and to obtain more food than males in foraging tasks^32^,^33^. In boldness, we expected no sex difference^34^. We expected differences in boldness between breeders and adult non-breeding helpers, because breeders and helpers differ in vigilance behaviour^35^.

## Results

### a. The social condition

Overall, marmoset responses showed consistent individual differences in behaviour in some, but not all, situations (Table 1). This indicates a mixture of consistency and plasticity in the measured behavioural responses. Temporal repeatability varied across experiments (intra-class correlation ICC(3,1) mean = 0.27, SD = 0.30). All but one of the measures were repeatable in at least two experiments, and all but one of the experiments (i.e. the novel environment) yielded some repeatable measures. There was no systematic difference in repeatability among the foraging tasks despite the possible differences in the cognitive challenge. Those responses that were temporally repeatable showed also moderate or high cross-situational consistency (Cronbach's alpha 0.50–0.96; Supplementary Table S1). Latency to solve the two more difficult foraging tasks, Bucket and Perspex, were negatively correlated. When those were excluded, the mean cross-situational consistency increased to Cronbach's alpha = 0.80.

The PCA of the consistent variables revealed a two-component structure (see Supplementary Material for further details on the analysis and diagnostics). The components explained 46.1% and 30.9%, respectively, of the variance (Table 2). Although there was partial conceptual overlap in the contents of the components, the first component included salient loadings of the responses in a predatory situation and was consequently labelled as Boldness; the second component included exploratory responses and interactions with the stimuli, and was therefore labelled as Exploration.

**Figure 1:**
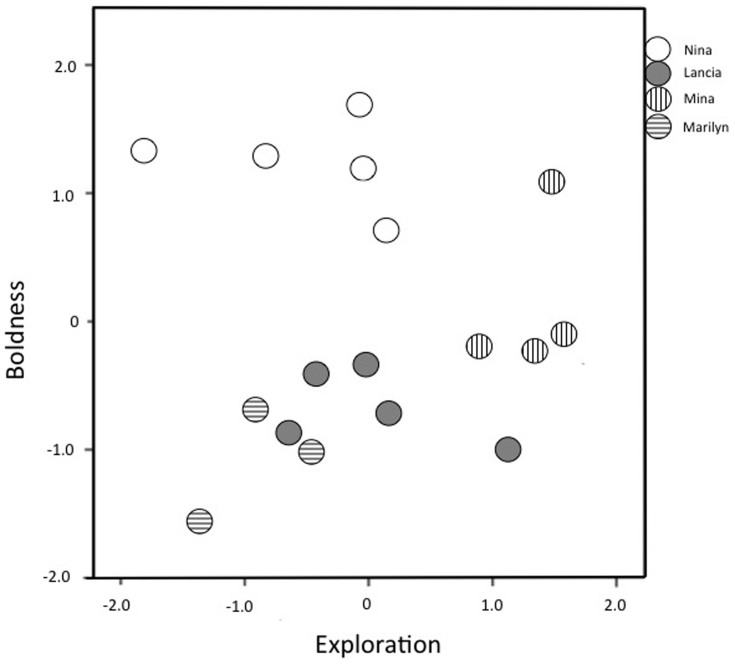
The component scores of the marmosets of the four groups. The groups are named after the breeding female.

There were no effects of sex or social role on either Boldness or Exploration scores (Boldness: sex F(5,11) = 1.02, *P* = 0.33; role: F(5,11) = 2.68, *P* = 0.13; Exploration: sex F(5,11) = 0.02, *P* = 0.89; role F(5,11) = 0.61, *P* = 0.45; Supplementary Table S2). However, group identity significantly predicted both boldness and exploration scores (Boldness: F(5,11) = 24.9, *P* < 0.0001; Exploration: F(5,11) = 8.09, *P* = 0.004). Thus, groups were significantly different from each other in both components, and the group members had similar personality scores (Fig. 1), which implies group-level personality.

**Figure 2:**
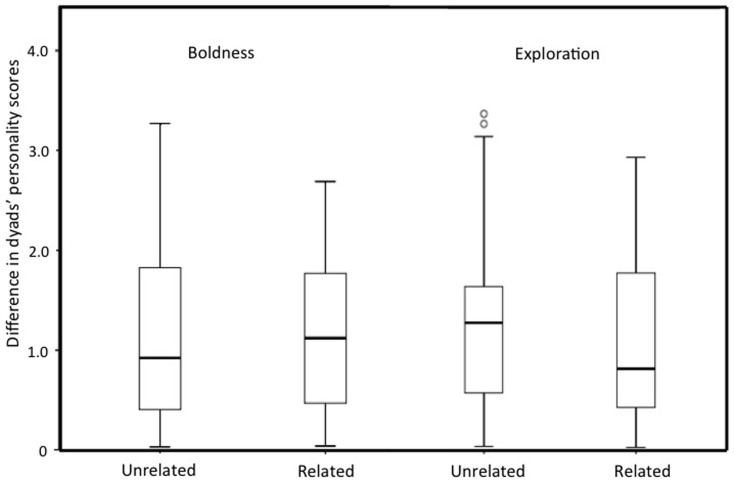
The dyadic difference of the boldness and exploration component scores in related (N = 53) and unrelated (N = 83) dyads. Difference between related and unrelated dyads in both traits n.s.

There was no difference between the scores of related and unrelated dyads in either trait (Boldness: *X*_unrelated_ = 1.14 (SD = 0.83), *X*_related_ = 1.18 (SD = 0.81); t = −0.32, *df* = 134, *P* = 0.75; Exploration: *X*_unrelated_ = 1.24 (SD = 0.78), *X*_related_ = 1.06 (SD = 0.83), t = 1.27, *df* = 134, *P* = 0.21; Fig. 2).

### b. The solitary condition

The social-to-solitary repeatability was low in the Bucket experiment (mean ICC = 0.06, SD = 0.19), and high in the Snake experiment (mean ICC = 0.60, SD = 0.19, table 1). This indicates that in the foraging task, individuals when alone did not behave as they had when with their group mates, whilst in the predatory situation they did. Although the exploratory behaviour was unrepeatable, we explored the variable structure with PCA. The two extracted, Varimax-rotated components explained 56.3 and 15.6% of the variance, respectively. The components were tentatively named Boldness and Exploration, noting that tendency to manipulate objects was associated with Boldness rather than with Exploration, as in the social condition (Table 2).

Boldness scores were again predicted by group identity (F (5,10) = 4.29, *P* = 0.039), indicating group-level personality, but not by sex or role (sex: F(5,10) = 0.67, *P* = 0.43; role: F (5,10) = 3.04, *P* = 0.11; Supplementary Table S2). Exploration scores showed no significant relationship with sex, role or group identity (sex: F (5,10) = 0.86, p = 0.38; role F(5,10) = 1.40, *P* = 0.26; group: F(5,10) = 2.06, *P* = 0.17, Supplementary Table S2).

## Discussion

Repeated experiments revealed that overall, marmoset personality is relatively plastic and subjected to social modification. The responses that showed temporal repeatability were also consistent across different experiments, indicating that individuals behaved consistently both over time and in different, conceptually similar situations. However, other responses showed low repeatability. The repeatable behaviours formed a structure of two independent components: predatory responses indicating “boldness”, and novelty-foraging responses indicating “exploration”. The latter also included persistence, i.e., continued exploration of an object without an immediate reward. The conceptual distinction between boldness and exploratory tendency was thus supported^36^,^43^, further strengthened by high cross-contextual consistency across novelty and foraging tasks, which decreased when the predatory context was included. Against our predictions, there were no differences in boldness or exploration tendency between sexes or between breeders and helpers.

We found considerable social modification in marmoset personality, which is consistent with the relatively high plasticity in personality. There was both short-term modification possibly by social facilitation, and a long-term effect producing sustained convergence of behaviour, both of which resulted in group-level similarity in behaviour. Exploration tendency was socially modified in the novel and problem solving situations, as the measures that were temporally and contextually repeatable in the social environment were not repeatable when individuals were tested alone. Moreover, individuals showed similarity in exploratory behaviour among group members in the social but not in the solitary situation. This suggests that the mechanism influencing exploratory behaviour is facilitation, which is not sustained when individuals are alone. Thus, individual exploratory behaviour is considerably influenced by group mates, which results in group-level personality sustained over a long time and different situations but not when individuals are alone.

Boldness appeared less susceptible to the short-term social effects, as individuals behaved consistently in social and solitary conditions. However, individuals showed group-level similarity in behaviour also in boldness, which persisted in the solitary condition. This suggests that the social environment affected also boldness but the mechanism is different than in exploratory behaviour. Convergence to a group-typical way of responding in threatening situations may occur early in life, or after immigration, or both. In our study, we cannot assess the actual mechanism underlying the convergence, when it occurs, or whether individuals converged towards a particular keystone individual, such as the breeding female, or towards a group mean. To understand the mechanism, longitudinal data on developmental effects and of emigrating individuals’ behaviour in a new group are crucial. The convergence in the vocal behaviour of pygmy marmosets after pairing^44^ indicates plasticity in mature individuals, but whether this extends to personality, remains to be assessed.

An alternative explanation to the findings is that distress in the solitary condition trumped the individuals’ usual behavioural tendencies in the foraging experiment. Unfortunately it was not possible to reliably quantify distress indicators in the experiments. However, distress can be expected to influence behaviour especially in a predatory context, but behaviour in the snake experiment remained consistent across the two conditions, suggesting that the distress explanation is unlikely. Another alternative explanation may be that in the solitary condition feeding competition was relaxed, allowing subordinate helpers freer access to the food-containing Bucket. However, this is unlikely because there was no difference between helpers’ and breeders’ scores in either condition, and there was no systematic increase in the breeders’ time spent in proximity of the bucket in the solitary condition (Supplementary Figure S1). A possible explanation for the low repeatability in several responses in the social condition is the presence of juveniles in the second, but not in the first, experimental round in three out of the four groups. Presence of dependent young may have changed the adults’ behaviour, especially in the predatory or foraging contexts. However, as there was no consistent pattern in the type of experiments to elicit unrepeatable responses, the presence of dependent young is unlikely to have been the main source of variation in adults’ behaviour. Nevertheless, variation in behaviour due to the presence of juveniles would agree with the main finding of this study, i.e., that marmoset personality is plastic and vulnerable to external, social influences. An alternative, non-social mechanism to explain the group-level similarity in behaviour is genetic canalization of personality traits that results in similar behaviour among relatives. However, it was found implausible for both boldness and exploratory tendency, because we found no difference in the scores of related and unrelated dyads.

Group-typical personality was thus most likely produced by social mechanisms. Regardless of whether these were short-term facilitation or long-term learning, in an everyday social context individuals within a group exhibited behaviour that is distinct to that group and differentiated it from other groups. Such “group personality” has to our knowledge not been described for mammals regarding personality traits (cf. group-level similarity in behaviour in cultural research^18^). Interestingly, similar evidence on group-specific personality scores was recently found in the common marmosets of Vienna University (Vedrana Slipogor pers. comm.), suggesting that group-level similarity in personality is a general phenomenon in marmosets. The finding is consistent also with group differences in social personality traits^20^ and “social styles”^21^ in chimpanzees.

A cautionary note on the strength of the evidence is due. The study was designed to address personality of all individuals in the population and the effect on the social environment on it. However, we found that group membership significantly predicted the individual personality scores, which we continued to explore. Unfortunately the number of groups is small, which allows only tentative interpretations of the mechanisms, functions, and implications of group-level similarity in personality. Nevertheless, we wish to suggest ideas to direct further work on “group personality”.

### Why “group personality”?

Social modification of personality that results in “group personality” is an important addition to the recent discussion on personality in a social environment^3^,^4^,^11^,^15^. One of the consequences is that in social organisms, group will have to be addressed as a potential level of analysis. Moreover, it raises the question of the ultimate function of group-level similarity in personality and the high level of plasticity in behaviour it requires.

We suggest two non-exclusive hypotheses for the ultimate function. Similarity of personality among group members may be beneficial in species with group-level cooperation and coordination. Similarity in behavioural tendencies among group members increases coordination and prosociality^45^,^46^,^47^, because similar affective states result in a cognitively inexpensive way of facilitating behavioural synchrony, contingency and reciprocity^48^,^49^. Closely cooperating individuals have similar personalities in humans^47^,^50^,^51^ and chimpanzees^52^. In marmosets, cooperation occurs at the group level, as all group members participate in active offspring care and proactive prosociality, which may be promoted by group-level similarity in personality.

Alternatively, group-level similarity in personality may occur as a byproduct of social learning. Habitual social learning requires tolerance to proximity and a high motivation to attend to and copy others’ activities. Therefore, if there are no particular costs to converging behaviours towards other group members’ behavioural style, social learning may result in behavioural convergence. In this case, we would expect group-level similarity in personality traits at least in all habitual social learners, and to concern traits associated with social learning, but not necessarily other traits.

The two hypotheses need not be exclusive. In habitual social learners, such as chimpanzees^53^ and marmosets^28^,^30^, group members may exhibit social modification of their social personality traits, such as the level of affiliation, gregariousness and social tolerance. However, social learning may not suffice to cause group similarity in other traits, including boldness and exploratory tendency, as these behaviours are less directly involved in interactions with the group mates. Moreover, if cooperation is mainly dyadic and there is a possibility to choose cooperation partners from a pool of group members, individuals can optimize the choice based on attributes like personality and effectiveness^52^,^54^, making group-level convergence in personality unnecessary. Marmosets, in contrast to chimpanzees, are cooperative at the group level^55^. Therefore, convergence to group-level similarity in personality traits may become adaptive due to its positive effects on cooperation.

The suggestion remains tentative until a formal assessment. That requires studies on personality in several species with different degrees of social learning and cooperation, and deliberate attention to group-level similarity in personality traits in multiple groups. In this study we have demonstrated that personality in a cooperatively breeding primate is subjected to both short-term and long-term social modification, which results in group-level similarity in fundamental personality traits. The next step is to understand the generality, mechanisms and adaptive function of such social effects.

## Methods

We assessed personality in 17 adult marmosets housed at the Primate Station of the Anthropological Institute of the University of Zurich. The subjects lived in 4 groups consisting of a breeding pair and 1–3 adult offspring. In addition, during the study three of the four groups had dependent young, which were not included in the study. Prior to and after the birth of the offspring, the adult females were treated with regular prostaglandin injections as contraception. One of the female helpers was expulsed one year after the onset of the study and was therefore not available for the solitary testing condition.

We conducted eight experiments in a social setting to test boldness, exploratory tendency, and persistence (Table 3). The experiment battery was repeated after 6 months in the social setting. One year later after the second experiment round, we tested the individuals in a solitary setting with two of the experiments. The experiment battery had high ecological validity: we simulated situations a group of marmosets might encounter in the daily life in a forest. Moreover, to improve comparability across species, the experiments were conceptualized after those done recently in chimpanzee personality research^22^.

The study complied with the national and international ethical guidelines of animal welfare and was approved by the Veterinary Office of the Canton of Zürich, license number 102/2012.

### Experimental protocol

In the social condition experiments, we tested the individuals in their own, permanent family groups in their home cages. Groups had no visual access to each other during the experiments. A group was subjected to one experiment per day. The experiments lasted for 10 minutes (all but predator model experiments) or 5 minutes (the predator model experiments), after which the experimental stimulus was removed. Two of the experiments were also tested in a solitary condition: the Bucket and the Snake (see below and Table 3). We limited the study to the two experiments to avoid potential distress due to the solitary condition, which lasted for the duration of the experiment (identical to the social condition), after which the subject was released back to its group. The Snake experiment was chosen as a predatory situation as it yields the most explicit measure of boldness (sensu^1^, Ref. 36 for a different interpretation); the Bucket was chosen for the estimated mid-level difficulty as a cognitively challenging foraging task. Each experiment was conducted once per subject. We tested the subjects alone in their home cage, with the rest of the subject's group within auditory, but not visual or tactile reach. In all other respects the protocol was identical with the social condition.

The experimental stimuli were in the categories of novelty, threat, and cognitive challenge in a foraging context (Table 3; for further information of the experimental details see Supplementary Material). In each category we conducted at least two different experiments to assess the cross-situational consistency of responses and to improve the validity of the assays^37^. The experiments in the novelty category included (i) a small novel object, a plastic beetle (1^st^ round) or spider (2^nd^ round), attached on the cage wall with tie ribs (“Novel Object S”), (ii) a large novel object, a plastic butterfly (green and yellow on 1^st^ round, brown and yellow and different wing patterns on 2^nd^ round), attached on the cage wall with tie ribs (“Novel Object L”), and (iii) novel environment, for which we rearranged the orientation of two tree branches in the home enclosure so that the travel route architecture was new in both rounds (“Novel Environment”). While the husbandry routine included occasional replacing and reorienting the branches, this occurred relatively seldom (ca. once a year). Therefore, we considered the rearranged branch structure to be perceived as novel. However, the results indicate this may not have been the case (see Table 1). The foraging tasks with a cognitive challenge included (i) a large, shallow cardboard box filled with floor bedding material and a large number of live mealworms, attached to an elevated platform (“Sandbox”); (ii) a round, 40 mm deep cardboard tube holding several small pieces of fruit and covered with triple-layered silk paper, attached to an elevated platform (“Bucket”); and (iii) a transparent Perspex box with two flap doors and a round opening, filled with small pieces of marshmallows, attached to an elevated platform (“Perspex”). All the foraging tasks and the objects were novel to the subjects. The predator model experiments included (i) a naturalistic looking, plastic red-and-black coral snake with a fishing line attached to its mouth. The snake was hidden under the floor bedding and pulled out slowly through the cage until it exited from a small hole in the front door (“Snake”). Pilot trials on other subjects indicated that the marmosets respond to the snake with a fearful reaction^38^,^39^ and (ii) a large, plastic, black silhouette of a generic raptor attached to a rope system above the outdoor enclosures (“Bird”). We simulated the bird flying over the home enclosure twice with ca. 30 sec. interval. The bird model had been used in an earlier study on other subjects where it has been shown to elicit fearful responses (Strasser & Burkart in prep.).

All experiments were filmed in the presence of the experimenter (SEK). Prior habituation had ensured that the subjects did not respond to her presence. All data were assessed from the videotapes. For each individual, we coded the variables (Table 3) individually with 1s resolution. 5% of videos were coded by a second person; inter-coder agreement was Pearson's r = 0.89.

### Data analyses

To improve normality and homogeneity of the data, individual scores of each variable were standardized per experiment paradigm and per round. Repeatability of the variables was calculated with the Intra-Class correlation^40^, which assesses the proportion of variance in the data due to differences between subjects. Repeatability was calculated for the responses between the first and the second social condition experiments (temporal repeatability), and for the means of the scores in the two social condition experiments and the corresponding scores obtained in the solitary condition (social to solitary -repeatability). Only if a variable passed the personality criterion of repeatability r > 0, we qualified it for further analyses on cross-situational consistency and structural analyses. This is crucial, because the fundamental expectation of personality is at least moderate repeatability. Cross-situational consistency was calculated with Cronbach's alpha for the variables among all the social condition experiments in which they were measured, as well as among the experiments of the same category (i.e., novelty, foraging task, and predatory situation). Unrepeatable variables (i.e. those indicated by F-test as r ≠ 0) were excluded. Cronbach's alpha was not assessed for the responses in the solitary experiments because no variables were repeatable in the solitary Bucket experiment.

The variables that had sufficient temporal and situational consistency were then merged as individual scores across experiments. The individual merged scores were entered into a principal component analysis (PCA). The number of extracted components was determined by the parallel analysis with 95% percentile rule^41^,^42^ and confirmed with a scree plot. This step was repeated for the behavioural scores from the solitary condition, except that the entered variables were not merged as there were only two experiments.

The assessment of group, sex, or social role influences on the obtained PCA component scores was done with general linear models. The component scores were the dependent variable and group identity, sex, and helper or breeder role were entered as fixed variables. The full models included interaction terms of sex x role, sex x group, role x group, and sex x role x group, and if non-significant, they were left out of the reduced model. The influence of genetic relatedness on scores was tested by calculating the absolute difference in every dyad's component scores and comparing the differences between related and unrelated individuals with t-tests (critical alpha level was corrected to p < 0.003). Relatedness was categorized with the relatedness coefficient r: r = 0 (N = 82), r = 0.125 (N = 9), r = 0.25 (N = 13), r = 0.5 (N = 32). Due to the bias to unrelated dyads, we pooled the groups and considered “related” to be all dyads with r > 0 (related N = 54, unrelated N = 82). Of the related dyads, 29 lived in different groups and 25 in the same groups. Of the unrelated dyads, 4 lived in the same group (i.e. the breeding pairs).

## Supplementary Material

Supplementary Information Supplementary Information

## Figures and Tables

**Table 1:** The repeatability as Intra-Class Correlation within the social condition (first and second round of experiments) and between the solitary condition and the social condition (mean of the two social condition rounds)

Experiment	Measure	ICC (3,1)	95%CI (lower, upper)	F, p
**Social condition**				
Novel obj. S	Latency approach	*0.31*	−0.18, 0.68	1.91, 0.10
	Latency touch	**0.45**	−0.03, 0.76	2.61, 0.03
	Duration proximity	**0.70**	0.35, 0.88	5.76, 0.001
	No. approaches	−0.04	−0.50, 0.43	0.92, 0.57
	Duration manip.	**0.81**	0.54, 0.93	9.25, <0.001
Novel obj. L	Latency approach	−0.11	−0.55, 0.37	0.80, 0.67
	Latency touch	0.03	−0.44, 0.49	1.07, 0.449
	Duration proximity	**0.78**	0.50, 0.92	8.21, <0.001
	No. approaches	*0.32*	−0.17, 0.69	1.96, 0.10
	Duration manip.	**0.79**	0.52, 0.92	8.62, <0.001
Novel env.	Latency approach	0.12	−0.37, 0.56	1.28, 0.32
	Latency touch	−0.01	−0.48, 0.46	0.98, 0.51
	Duration manip.	−0.11	−0.55, 0.38	0.80, 0.67
Sandbox	Latency approach	−0.21	−0.62, 0.28	0.65, 0.81
	Latency touch	−0.16	−0.59, 0.33	0.72, 0.74
	Duration proximity	**0.49**	0.02, 0.78	2.89, 0.02
	No. approaches	**0.48**	0.01, 0.77	2.83, 0.02
	Duration manip.	0.03	−0.44, 0.49	1.06, 0.45
	No. items cons'd	0.15	−0.35, 0.58	1.34, 0.28
Bucket	Latency approach	**0.47**	0.01, 0.77	2.79, 0.02
	Latency touch	**0.50**	0.04, 0.78	2.98, 0.02
	Duration proximity	0.14	−0.35, 0.57	1.34, 0.29
	No. approaches	0.13	−0.36, 0.56	1.29, 0.31
	Duration manip.	0.27	−0.23, 0.65	1.73, 0.14
	Latency solve	**0.76**	0.45, 0.92	7,22, <0.001
	No. items cons'd	0.23	−0.27, 0.63	1,60, 0.18
Perspex	Latency approach	**0.45**	−0.02, 0.76	2.66, 0.03
	Latency touch	**0.44**	−0.03, 0.78	2.59, 0.03
	Duration proximity	−0.30	−0.67, 0.20	0.54, 0.88
	No. approaches	**0.42**	−0.07, 0.74	2.42, 0.04
	Duration manip.	−0.10	−0.55, 0.38	0.81, 0.66
	Latency solve	**0.64**	0.25, 0.86	4.62, 0.002
	No. items cons'd	0.27	−0.23, 0.65	1.73, 0.14
Bird	Latency approach	−0.25	−0.64, 0.25	0.60, 0.84
	Duration proximity	0.13	−0.37, 0.56	1.29, 0.31
	No. approaches	*0.33*	−169, 0.69	1.96, 0.09
Snake	Latency approach	**0.69**	0.32, 0.87	5.40, 0.001
	Duration proximity	**0.63**	0.23, 0.85	4.37, 0.003
	No. approaches	**0.50**	0.04, 0.78	2.96, 0.02
**Solitary condition**				
Bucket	Latency approach	0.30	−0.22, 0.68	1.84, 0.13
	Latency touch	0.20	−0.31, 0.62	1.51, 0.22
	Duration in proximity	0.17	−0.34, 0.60	1.40, 0.26
	No. approaches	−0.20	−0.62, 0.31	0.67, 0.78
	Duration manip.	−0.08	−0.54, 0.42	0.86, 0.61
	Latency solve	−0.02	−0.50, 0.47	0.97, 0.53
Snake	Latency approach	**0.40**	−0.11, 0.74	2.31, 0.05
	Duration proximity	**0.78**	0.48, 0.92	8.13, <0.001
	No. approaches	**0.62**	0.19, 0.85	4.20, 0.004

ICC (3,1) values are given as consistency agreement and single correlation. **Bold** typeface signifies repeatability significantly above 0, *italics* a trend at significance level 0.05 < p ≤ 0.10 by F-test.

**Table 2:** Principal components and loading scores of behaviours after Varimax rotation in the social and the solitary conditions. The behavioural scores were merged only in the social condition. h2 = variable communality

Social condition	Boldness	Exploration	h2	Solitary condition	Boldness	Exploration	h2
Latency approach Snake	**−0.93**	0.04	0.87	Latency approach Snake	**−0.77**	−0.29	0.67
No. approaches Snake	**0.93**	0.11	0.88	No. approaches Snake	**0.85**	0.14	0.74
No. approaches (merged)	**0.80**	−0.04	0.64	Duration proxim. Snake	**0.85**	0.18	0.75
Latency solve Bucket	**−0.70**	**−0.51**	0.74	Duration manip. Bucket	**0.63**	0.30	0.48
Duration proxim. (merged)	−0.26	**0.91**	0.90	Duration proxim. Bucket	**0.60**	**0.65**	0.78
Duration manip. (merged)	−0.07	**0.85**	0.73	Latency solve Bucket	−0.36	**−0.72**	0.65
Latency touch (merged)	−0.27	**−0.73**	0.60	Latency approach Bucket	−0.15	**−0.91**	0.84
Latency approach (merged)	**−0.46**	**−0.72**	0.73	Latency touch Bucket	−0.19	**−0.90**	0.85
Eigenvalue	3.69	2.40		Eigenvalue	4.51	1.25	

**Table 3:** The experimental assays, the targeted traits, and the measured variables

Experiment	Targeted traits	Measured variables
1. Novel object, small	Exploration (Boldness)	Latency to approach, latency to touch, no. approaches, time in proximity, time used manipulating
2. Novel object, large	Exploration (Boldness)	Latency to approach, latency to touch, no. approaches, time in proximity, time used manipulating
3. Novel environment	Exploration	Latency to touch the first new part, latency to touch the second new part, duration of manipulating
4. Sandbox	Exploration Persistence	Latency to approach, latency to touch, no. approaches, time in proximity, time used manipulating ( = searching), no. items consumed
5. Bucket	Exploration Persistence Problem-solving	Latency to approach, latency to touch, no. approaches, time in proximity, time used manipulating ( = searching) until the first retrieved item, no. items consumed
6. Perspex	Exploration Persistence Problem-solving	Latency to approach, latency to touch, no. approaches, time in proximity, time used manipulating ( = searching), no. items consumed
7. Snake	Boldness	Latency to approach, latency to touch/sniff, no. approaches, duration in proximity
8. Bird	Boldness	Latency to approach, no. approaches, duration in proximity
