# Supplementary material for: Common marmosets show social plasticity and group-level similarity in personality
Source: Sci Rep. 2015 Mar 6;5:8878. doi: 10.1038/srep08878 (PMC5155412; doi:10.1038/srep08878)
Supplement: Supplementary Information [file srep08878-s1.pdf]

Supplementary Material

Common marmosets show social plasticity and group-level similarity in personality

Sonja E. Koski*, Judith M. Burkart

Anthropological Institute and Museum

University of Zürich

Winterthurerstrasse 190, CH-8057 Zürich, Switzerland

Methods

Subjects and housing

We tested 17 adult marmosets in the Primate Station facility of Zürich University. All individuals were born in the facility and reared by their natural parents in the family groups. They were housed in large indoor-outdoor enclosures comprising one or several basic units (1 x 0.75 x 1.7 m indoors; 2.75 x 1.7 or 2.5 x 2.4 m outdoors) that include ropes, branches, and other enrichment devices, and were covered with natural bedding material. Both indoor and outdoor enclosures had heating lamps. Subjects almost continuously had free access to both enclosures, except during the necessary husbandry routines, at outside temperature < 5°C and at nights. They were fed three times a day with a diet of carbohydrate-rich mush enriched with vitamins and minerals, as well as fruit, vegetables, gum, insects, boiled egg, and nuts. Water was available ad libitum.

Over the 18-month study time, the groups were stable in their dynamics. Between the two rounds of the social experiments, in summer 2012, three out of four groups had twins, which did not result in any observable increase in aggression or decrease in cohesion. One of the groups (Lancia, incl. 3 adult helpers) experienced a short-term upheaval approx. 2 months before the solitary condition experiments, which led to an expulsion of a female helper. After the female was removed, the social dynamics were returned to peaceful.

The groups did not continue breeding after the birth of the offspring in summer 2012. Pregnancies were prevented by a regular prostaglandin treatment of mature females.

Experimental protocol

Experiments were approved by the Veterinary Office of the Canton of Zürich, licence number 102/2012. The social condition experiments were conducted in March-April 2012 (round 1) and October-November 2012 (round 2). The solitary condition experiments were conducted one year later, in October 2013.

Experiments were timed after a feeding session so that the subjects were not hungry. The experiments were conducted in the groups' home enclosures. The stimuli were prepared without individuals' visual, olfactory or auditory access. In the social setting experiments, the whole group was simultaneously released to the enclosure and the order of arrival was filmed. The subjects were free to interact with the stimulus for 10 min (or 5 min in the case of the predator models), after which the experiment ended and the stimulus was removed from the enclosure. The order of experiments was randomized, however, the two predator models were never presented on consecutive days. Only one experiment was conducted per day per group. To avoid priming individuals with alarm calls from neighbouring groups, the experiments involving a predator model were conducted with only one group per day. To further minimise stress as a possible confounder, all stimuli except the predator models were presented in an elevated location (height 90-150 cm from ground) on the wall or a familiar platform[1].

In the solitary setting, individuals were called to the home cage by name whilst holding others in another enclosure. The test subject had auditory, but no visual or tactile access to its group members. The testing duration was the same as in the social condition: 10 min in the Bucket experiment and 5 min. in the Snake experiment

(see below). After the experiment, the subject was immediately released back to its group.

Experiment details

1. **Novel object, small**. We attached a plastic, purple and yellow toy beetle (1st experiment round: 45 x 25mm) or a plastic, red and black toy spider (2nd experiment round: 55 x 20 mm) on the wall of the enclosure using tie-ribs. Marmosets had never seen either of the objects, but were familiar with tie-ribs. The targeted trait was exploratory tendency as a response to a small, hence presumably not threatening, naturalistic-looking novel object. Subjects' approaches to, and leaves from, the proximity (defined as 20 cm radius of the object), and all interactions with the object were recorded.

2. **Novel object, large**. We attached a colourful plastic toy butterfly (1st experiment round: green and yellow 200 x 120 mm; 2nd experiment round: brown and yellow 180 x 150 mm) on the wall of the enclosure using tie-ribs. The subjects had never seen the butterflies before. The targeted trait was exploratory tendency as a response to a large (nearly the size of a marmoset torso) and hence possibly threatening, naturalistic-looking novel object. Subjects' approaches to, and leaves from, the proximity (defined as 20 cm radius of the object), and all interactions with the object were recorded.

3. **Novel environment.** We rearranged the orientation of two existing tree branches in the home enclosure so that the travel routes in the enclosure were altered. The branches were large and inter-supporting, so the rearrangement necessarily also changed several attachment points. At both experiment rounds, the composition of the branches was novel. The regular husbandry routine occasionally included adding new branches, which necessarily changed the branch orientation. However, as this occurred only ca. once a year, we considered the change of the travel routes to be novel enough to elicit exploratory behaviour. The targeted trait was exploratory tendency in a novel environment that might occur in the home territory, e.g. after a storm. We recorded the latencies to approach the first and the second new branch, respectively, and the duration exploring (scrutinizing, sniffing, marking) the new branches.

4. **Sandbox**. We buried a large number of live mealworms (ca. 10/individual) in a shallow cardboard box of 39 x 29 x 12 cm filled with bark-like floor bedding. The box was attached to an elevated platform, which was familiar to the subjects. The subjects had never been exposed to such boxes, although they were familiar with digging mealworms out of a matrix. The targeted traits were exploratory behaviour and persistence in a cognitively easy foraging context that requires continuing exploration after the first gain. We measured both the initial neophilic response and the continuing willingness to persist with searching. Subjects' approaches to, and leaves from, the proximity (defined as 20 cm radius of the object), and foraging efforts (searching time and number of items consumed) in the box were recorded.

5. **Bucket**. We attached a round cardboard "bucket" (depth 40 mm, diameter 105 mm) covered with triple-layered silk paper attached to an elevated platform. Inside the covered bucket was a large quantity of fruit (pieces of banana, grape, apple, melon).

The object was novel for the monkeys. The targeted traits were exploratory behaviour and persistence in a moderately difficult cognitive challenge. To gain access to the rewards, subjects had to break through the silk paper, i.e. successful foraging required one-off problem solving. We assessed both the initial response to a novel object in a foraging context, and the problem-solving latency. We recorded subjects' approaches to, and leaves from, the proximity (defined as 20 cm radius of the object), efforts to gain access to the food until the first item retrieved (i.e. time manipulating the bucket or silk paper with hands or teeth, sniffing or otherwise exploring the bucket), and the number of items consumed.

6. **Perspex**. We attached a transparent Perspex box of 120 x 80 x 95 mm on an elevated platform. The box had three ways to access the rewards: a hole of 33 mm radius, an upward-opening flap-door, and a downward-opening flap-door (see also[2]). Inside the box was a large quantity of very small pieces of marshmallow. Subjects' liking of marshmallows had been established earlier; all were interested in them. The object was novel for the monkeys. The targeted traits were exploratory behaviour and persistence in a demanding cognitive challenge. Previous experiments on other subjects had shown the box to be cognitively challenging. To gain access to the rewards, subjects had to discover at least one of the access routes and maintain it, as the flap-doors did not stay open unless held. We measured the initial response to a novel object in a foraging context, problem-solving latency, and persistence in efforts to repeatedly gain access to the rewards. We recorded subjects' approaches to, and leaves from, the proximity (defined as 20 cm radius of the object), efforts to gain access to the food (i.e. sniffs, manipulation with hands, teeth, or head of any part of the object), and the number of items consumed. The manipulation duration was recorded throughout the experiment because the subjects did not appear to remember the solution after the first successful retrieval.

7. **Predator model: snake**. A naturalistic looking model of a red-and-black coral snake (length 100 cm) with a fishing line attached to its mouth was hidden under the floor bedding in the enclosure. Pilot experiments with a different set of animals had shown that marmosets react fearfully to a snake model, despite having never encountered a snake before. When the group had been released to the enclosure, we waited until they were engaged in normal activities and showed no signs of anxiety or fear (minimum 2 min). We then pulled the snake until it was visible, and continued to pull it slowly towards the front of the cage where the snake exited through a small hole on the door and was taken out of sight. The transfer of the snake from the back to the front of the cage took 5 min. The targeted trait was boldness, i.e. responses to a naturalistic predator threat. We recorded all approaches to, and leaves from, the proximity (defined as 50 cm radius), and touches and sniffing of the snake.

8. **Predator model: Bird.** We simulated a large bird of prey flying over the home enclosure. The model was a 45 x 75 cm plastic, black silhouette of a generic raptor attached to a rope system above the outdoor enclosures. By pulling the ropes we simulated the bird flying from one side of the enclosure to the other side, and after ca. 30 sec to the reverse direction. Earlier research on other subjects showed that the model elicited fearful responses. Moreover, the subjects frequently saw raptors flying above their outdoor enclosure to which they responded with tsk-calls. Similarly to the snake experiment, we waited until the group engaged in normal activities and showed no signs of anxiety before starting the experiment. Only the experimental group saw

the stimulus, other groups were held in their indoor enclosures with no visual access to outdoors. The targeted trait was boldness, i.e. responses to a naturalistic predator threat. We recorded all approaches to, and leaves from, the proximity of the model, defined as the upper 20cm of the outdoor cage.

All experiments were filmed in the presence of the experimenter (SK). Prior habituation had ensured that the subjects did not respond to her presence. All data were assessed from the videotapes. We filmed 70 cm around the experiment stimulus, as in the social condition it was not possible to keep every individual in the camera frame at every moment. In addition, the experimenter quietly narrated the events and identified individuals during the experiment. Latencies to approach and to touch a stimulus were calculated from the exact moment the subject entered the enclosure. An approach counted as a separate incident when the subject left the proximity of the stimulus for at least three seconds.

RESULTS

Repeatability of the variables in the two rounds of experiments in the social condition was average: ICC = 0.27 (SD = 0.30). However, there was large variation in repeatability so that variables were repeatable in some, but not in other experiments. Social-to-solitary repeatability was moderate to high in the Snake experiment but low in the Bucket experiment. In the latter, only the latency to approach the stimulus showed moderate repeatability (ICC= 0.30).

To assess whether inconsistency of exploratory behaviour was due to dominant individuals hindering subordinates' access to the Bucket in the social, but not in the solitary condition, we assessed the individual scores in the two conditions by drawing individual reaction norm plots. Supplementary Figure S1 shows the duration of time in proximity of the bucket for each individual in the two conditions (ICC = 0.17). There was no consistent increase in the time in proximity by the subordinate helpers in the solitary condition. The other unrepeatable variables showed similar absence of a role-determined pattern (plots not depicted). Thus, competitive exclusion by dominant breeders was unlikely to explain the inconsistency.

The cross-situational consistency in the social condition was high in nearly all behaviours that were temporally repeatable. Supplementary Table S1 shows the consistency values and the combinations used for the merged individual scores. In the solitary condition experiments, behavioural responses in the snake and the bucket experiments were not merged.

The merged individual scores of the temporally and situationally consistent variables from the social condition experiments were analysed with PCA. Latency to solve Perspex was removed due to poor communality and weak loading, leaving eight variables in the final analysis. Based on the parallel analysis (95th percentile rule[3]), two components' eigenvalues exceeded chance level. Two components were extracted and the solution was Varimax rotated. Diagnostics supported good sampling adequacy (KMO=0.59; Bartlett's test of sphericity p=<0.0001). The two components explained 46.1% and 30.9%, respectively, of the variance. Although there was partial conceptual overlap in the contents of the components, the first component included responses in a predatory situation and was consequently labelled Boldness, whilst the second component included exploratory responses and interactions with the stimuli, and was therefore labelled as Exploration.

In spite of the low repeatability of behaviours in the Bucket experiment in the solitary condition, we conducted a PCA also on the variables in the solitary condition

experiments to assess whether the behaviours would show similar structure as found in the social condition. The number of approaches to the bucket and the number of items consumed were excluded due to poor communalities and low loadings. The sampling adequacy was moderate (KMO = 0.44; Bartlett's test of sphericity $p < 0.0001$), so the results are to be interpreted with some caution. Parallel analysis revealed that only one component exceeded the eigenvalue derived by randomizing. However, as the second component's eigenvalue was close to the mean derived by randomizing and the first set of results gave theoretical support for two components, we forced a two-component solution[4], which was Varimax rotated. The components explained 56.3 and 15.6% of the variance, respectively. The components were tentatively named Boldness and Exploration, with the note that tendency to manipulate objects was associated with Boldness rather than with Exploration, as in the social condition experiments.

Finally, we assessed with general linear models whether sex, breeder/helper role, or group identity predict the component scores in social and solitary conditions. The conditions were tested with separate models, because the PCAs included slightly different variables (social condition: merged scores, multiple experiments, strong diagnostic criteria for variable inclusion; solitary condition: two experiments only, unmerged scores, weaker PCA solution). The two- and three-way interactions were included in the full models, and if insignificant, they were left out of the reduced models. Supplementary Table 2 shows the effects of the predictors in the reduced models.

Supplementary Table S1. Cross-situational consistency of behaviours measured in the experiments in the social condition. Bold typeface signifies the combination of experiments used to derive a merged individual score of the variable.

Variable	Experiments	Cronbach's alpha
Latency approach	Bucket & Perspex	0.90
	**Bucket & Perspex & Novel object S**	0.73
	Bucket & Perspex & Novel object S & Snake	0.23
Latency touch	**Bucket & Perspex & Novel object S**	0.52
Duration proximity	Novel object S & Novel object L	0.86
	**Novel object S & Novel object L & Sandbox**	0.90
	Novel object S & Novel object L & Sandbox & Snake	0.50
No. Approaches	**Sandbox & Perspex**	0.76
	Sandbox & Perspex & Snake	0.80
Duration manipulation	**Novel object S & Novel object L**	0.96
Latency solve	Bucket & Perspex	-0.24

Supplementary Table S2. The effects of sex, role and group identity on the component scores in the (a) social and (b) solitary condition.

	**Boldness**			**Exploration**		
a.	Beta ± SE	F	*P*	Beta ± SE	F	*P*
Sex		1.02	0.33		0.02	0.89
female	0.21 ± 0.21			-0.05 ± 0.33		
male*	--			--		
Role		2.68	0.13		0.61	0.45
breeder	-0.34 ± 0.21			-0.26 ± 0.33		
helper*	--			--		
Group		**24.93**	**0.00003**		**8.09**	**0.004**
Nina	2.22 ± 0.31			0.32 ± 0.49		
Lancia	0.28 ± 0.32			0.90 ± 0.50		
Mina	1.12 ± 0.32			2.20 ± 0.51		
Marilyn*	--			--		
b.						
Sex		0.67	0.43		0.86	0.38
female	0.29 ± 0.36			-0.44 ± 0.48		
male*	--			--		
Role		3.04	0.11		1.40	0.26
breeder	-0.62 ± 0.36			0.57 ± 0.48		
helper*	--			--		
Group		**4.84**	**0.03**		2.06	0.17
Nina	1.61 ± 0.52			1.65 ± 0.69		
Lancia	0.87 ± 0.54			1.12 ± 0.72		
Mina	1.84 ± 0.54			0.69 ± 0.72		
Marilyn*	--			--		

* set as the reference variable

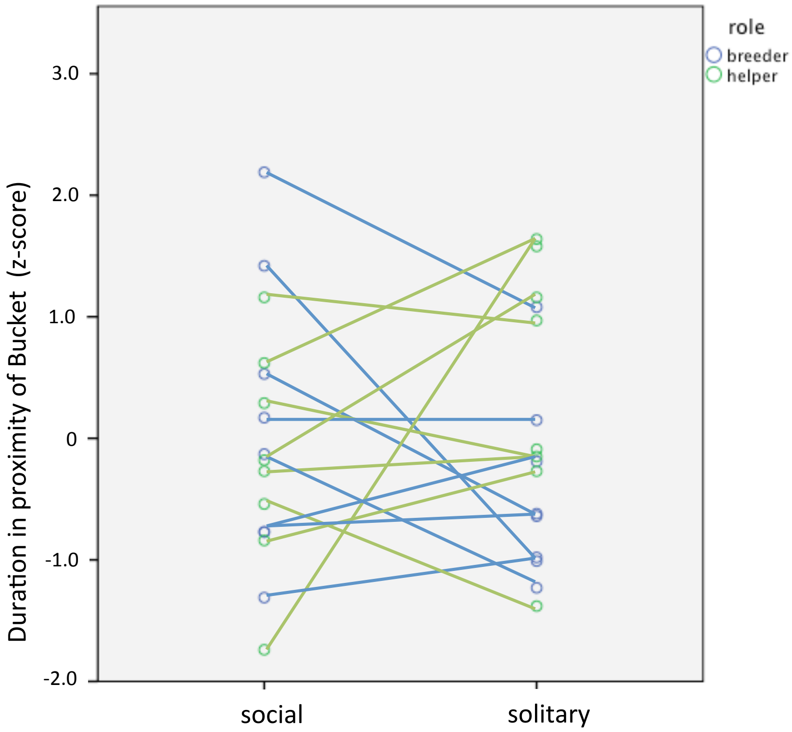

Supplementary Figure S1. Individual behavioural responses in the social and solitary test condition for the duration of time spent in proximity of the Bucket.
Blue lines depict the breeders and green lines depict the adult helpers.

References

1. Majolo, B., Buchanan-Smith, H. M., & Bell, J. Response to novel objects and foraging tasks by common marmoset (Callithrix jacchus) female pairs. *Lab Anim*. **32**, 40–46. (2003).
2. Burkart, J. M., Strasser, A., & Foglia, M. Trade-offs between social learning and individual innovativeness in common marmosets, Callithrix jacchus. *Anim. Behav*. **77**, 1291–1301. (2009).
3. Glorfeld L.W. An improvement on Horn's parallel analysis methodology for selecting the correct number of factors to retain. *Educ. Psychol. Measurement* **55**, 377-393. (1995).
4. Budaev, S. V. Using Principal Components and Factor Analysis in Animal Behaviour Research: Caveats and Guidelines. *Ethology* **116,** 472–480. (2010).
